# IL-11 mediates the Radioresistance of Cervical Cancer Cells via the PI3K/Akt Signaling Pathway

**DOI:** 10.7150/jca.56185

**Published:** 2021-06-01

**Authors:** Ruige Sun, Chunli Chen, Xinzhou Deng, Fengqin Wang, Shimao Song, Qiang Cai, Jincheng Wang, Te Zhang, Mingliang Shi, Qing Ke, Zhiguo Luo

**Affiliations:** 1 Postgraduate Training Basement of Jinzhou Medical University, Taihe Hospital, Hubei University of Medicine, Shiyan City, Hubei Province 442000, China.; 2 Department of Clinical Oncology, Taihe Hospital, Shiyan City, Hubei Province 442000, China.; 3 Graduate School of Guangxi Medical University, Nanning, Guangxi, 530021, China.; 4 Institute of Medicine and Nursing, Hubei University of Medicine, Shiyan City, Hubei Province 442000, China.; 5 Biomedical Research Institute, Hubei University of Medicine, Shiyan City, Hubei Province 442000, China.; 6 Department of Gastroenterology, Zhushan People's Hospital, Shiyan City, Hubei Province 442000, China.

**Keywords:** cervical cancer, radioresistance, IL-11, radiotherapy

## Abstract

Cervical cancer is one of the most common malignant tumors in the female reproductive system. Radioresistance remains a significant factor that limits the efficacy of radiotherapy for cervical cancer. Interleukin‐11 (IL-11) has been reported to be upregulated in various types of human cancer and correlate with clinical stage and poor survival. However, the exact effects and mechanisms of IL-11 in the radioresistance of cervical cancer have not yet been defined. In this research, TCGA databases revealed that IL-11 expression was upregulated in cervical cancer tissues and was associated with clinical stages and poor prognosis in cervical cancer patients. We discovered that IL-11 concentration was significantly upregulated in radioresistant cervical cancer cells. Knocking down IL-11 in Hela cells could reduce clonogenic survival rate, decrease cell viability, induce G2/M phase block, and facilitate cell apoptosis. In contrast, Exogeneous IL-11 in C33A cells could upregulate clonogenic survival rate, increase cell viability, curb G2/M phase block, and cell apoptosis. Mechanistic investigations showed that radioresistance conferred by IL-11 was attributed to the activation of the PI3K/Akt signaling pathway. Altogether, our results demonstrate that IL-11 might be involved in radioresistance, and IL-11 may be a potent radiosensitization target for cervical cancer therapy.

## Introduction

Cervical cancer is one of the most common malignancies in women worldwide, especially in less developed countries 1. Radiotherapy is considered as a primary treatment for cervical cancer patients 2. However, some cervical cancer patients are resistant to radiotherapy, which frequently lead to tumor recurrence and metastasis 3, 4. Therefore, identifying the underlying mechanisms of radioresistance is essential to improve the survival rate of patients.

As we knew, inflammatory response plays a pivotal role in different stages of tumor progression, in which cytokines are thought to bridge the gap between inflammation and cancer 5. Previous studies indicated that increased expression of cytokines, such as the Interleukin‐6 (IL-6) family of cytokines, is strongly associated with tumor growth, progression, and recurrence 6, 7. IL-11, a member of the IL-6 family of cytokines, shares a signal‐transducing receptor subunit (GP130) of this family. IL-6 and IL-11 compete for interaction with GP130, executing biological functions by depending on the expression patterns of their respective receptor subunits 8. IL-11 plays a vital role in tumorigenesis and cancer progression 9. For example, IL-11 promoted cell migration and invasion in non-small cell lung adenocarcinoma 10. And it has also been documented that downregulation of L-11 inhibits the proliferation, migration and invasion in esophageal squamous cell cancer 11. Nevertheless, it is still unclear that the exact roles and underlying mechanisms of IL-11 in cervical cancer radioresistance.

In the present study, we first determined the expression of IL-11 in the cervical cancer radiosensitive and radioresistant cells. Secondly, we investigated the function of IL-11 in radioresponse of cervical cancer cells. Finally, the underlying molecular mechanisms by which IL-11 is involved in radioresistance were discussed. Our findings suggested that targeting IL-11 might be a promising therapeutic option for the treatment of radioresistance in cervical cancer patients.

## Materials and Methods

### TCGA Data Analysis

The IL-11mRNA expression profiles in cervical cancer tissues and normal cervical tissues were obtained from The Cancer Genome Atlas (TCGA) database (https://tcga-data.nci.nih.gov). The clinical stage data of the cervical cancer patients were also obtained from the TCGA database. The prognostic value of IL-11 was evaluated in cervical cancer cohort from TCGA database through using Kaplan-Meier method and log-rank test.

### Cell lines and reagents

The cervical cancer cell lines HeLa, SiHa, C33A, CaSki, Ect-1 were purchased from the cell library of the Shanghai Institute of Life Sciences (the Chinese Academy of Science). Both cell lines were cultured in DMEM (Gibco BRL, Carlsbad, CA, USA) supplemented with 10% fetal bovine serum and maintained at 37 °C in a humid atmosphere with 5% CO_2_. Recombinant human IL-11 (rhIL-11) (R&D Systems, Minneapolis, MN, USA) was diluted in cell culture medium.

### Cell transfection

To establish stable IL-11 knockdown cell lines, three IL-11 shRNA and one negative control shRNA were designed and chemically synthesized by GenePharma (Shanghai, China). The shRNA sequences used were: sh-1, 5′-CAGAGGAGCAGACCGCAAAG-3′; sh-2, 5′-TGACGCTGAGGGACAAATT-3′; sh-3, 5′-TGAGCCTGGCCAGATACAG-3′; and negative control, 5′-UUGAUUGAUAGCCUGGACT-3′. shRNAs were transfected using Lipofectamine 3000 (Invitrogen, Carlsbad, CA, USA) according to the manufacturer's protocol.

### Quantitative real-time PCR (qRT-PCR)

Total RNA was extracted from cells using TRIzol reagent (Invitrogen), and 1ug of total RNA was reverse transcribed to cDNAs using a Reverse Transcription Kit (Takara, Dalian, China). Quantitative real-time PCR was performed using SYBR Green Real-Time PCR Master Mix kit (Toyobo, Osaka, Japan). Primer sequences for PCR assays designed were as follows: IL-11 forward, 5′-CGAGCGGACCTACTGTCCTA-3′; reverse, 5′-GCCCAGTGAAGTGTCAGGTG-3′; GAPDH forward, 5′-CCAACCGCGAGAAGATGA-3′; and reverse 5′-CCAGAGGCGTACAGGGATAG-3′. The 2^-ΔΔCt^ method was used to identify the relative mRNA expression.

### ELISA assay

IL-11 concentration in cell culture supernatant was determined by the Human IL-11 Immunoassay ELISA kit (Dakewei Biotech Company, Shanghai, China) following the manufacturer's instructions.

### Colony formation assay

For colony formation assays, different numbers of cells were seeded into 6-well plates (1000/well), and irradiated the next day with 0, 2, 4, 6, 8 Gy at room temperature using an X-ray irradiator (X-RAD 320, Precision X-ray) at 320 kV, 10 mA with a 2-mmaluminum filter, and the dose rate was 2 Gy/min. After incubating at 37 °C for 14 days post-irradiation, cells were washed twice with PBS, fixed for 15 minutes with 100% methanol, and stained for 15 minutes with 0.1% crystal violet (Sigma, St. Louis, MO, USA). Colonies containing 50 cells or more were counted. The surviving fraction (SF) was estimated by using the equation (mean colony counts)/(cells plated)×(plating efficiency), where the plating efficiency (PE) was calculated as (mean colony counts)/(cells plated for irradiated controls). Experiments were performed in triplicate.

### Cell viability assay

Exponentially growing cells were seeded at a concentration of 5×10^3^ cells per cell. After culturing at 37 °C overnight, the cells were exposed to various doses of X-ray irradiation (0, 2, 4, 6 and 8 Gy). Moreover, after irradiation treatment, the cells were incubated for an additional 24 h, the cell viability was evaluated using the Cell Counting Kit-8 (Beyotime, Jiangsu, China) following the manufacturer's instructions. The absorbance was detected at 450 nm using a microplate reader (Bio-Rad, Gaithersburg, MD, USA).

### Cell cycle and apoptosis analysis

Cells were treated with the indicated doses of IR. After 24h, the cell cycle and apoptosis assay were determined. The cell cycle was analyzed using the Cell Cycle Kit (Beyotime) according to the manufacturer's instructions. Cultured cells were collected by trypsinization and washed twice in cold PBS. Then the cells fixed in 70% ethanol overnight at 4 °C and washed twice with cold PBS. Next, the cells were resuspended in RNase A/PI staining solution and incubated in the dark at 37 °C for 60 minutes. For apoptosis assays, floating and adherent cells were collected by trypsinization and washed twice with cold PBS. The cells were resuspended in 1×binding buffer at a concentration of 1×10^5^ cells/mL, and stained for 15 minutes at room temperature in the dark using the AnnexinV/PI double staining Kit (Beyotime) according to the manufacturer's protocol. The cell cycle distribution and apoptosis rate were measured with flow cytometry (Becton-Dickinson, Mountain View, CA, USA).

### Western blot analysis

Protein extraction from whole cell lysates was performed using RIPA buffer (Beyotime, Jiangsu, China). The total protein was quantified using a BCA Protein Assay Kit (Beyotime). Equal amounts of protein (30 µg) were isolated using 10% SDS-PAGE and transferred onto PVDF membranes (Millipore, Billerica, MA, USA). Membranes were blocked with 5% non-fat dried milk at room temperature. The following primary antibodies were used in this study, anti-phospho-STAT3 (Y705) (ab76315; 1:1000, Abcam), anti-STAT3 (ab68153; 1:1000, Abcam). anti-phospho-Erk (#4370S; Cell Signaling Technology, 1:2000), anti-Erk (#4695S; Cell Signaling Technology, 1:1000), anti-phospho-Akt (Ser473) (#4060; Cell Signaling, 1:1000), anti-Akt (#4691; Cell Signaling Technology, 1:2000), anti-Bcl-2 (#15071T; Cell Signaling Technology, 1:1000), anti-Bax (#5023S; Cell Signaling Technology, 1:1000), anti-Bcl-xl (#2762S; Cell Signaling Technology, 1:500), anti-GAPDH (BL006B; biosharp, 1:1000). The protein bands were visualized by employing an ECL kit (Thermo Fisher Scientific, Rockford, IL, USA).

### Statistical analysis

Each experiment was performed at least three times. All statistical analysis was performed using GraphPad Prism 8.0 software (La Jolla, CA, USA). All results were expressed as the mean ± SD. Student's t-test and one-way ANOVA were used for comparison among groups. *P*<0.05 was considered significant.

## Results

### IL-11 is overexpressed and associated with poor prognosis in cervical cancer

**Figure 1:**
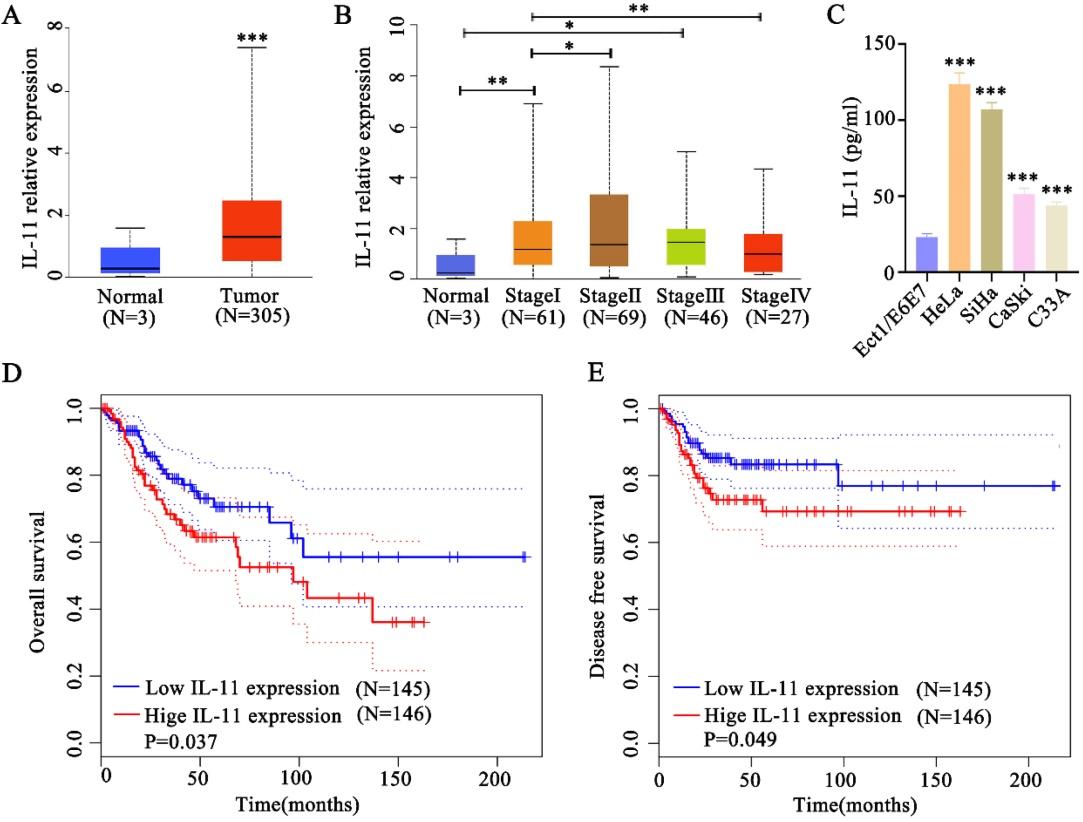
** IL-11 expression is increased in cervical cancer and correlates with poor prognosis.** (A) IL-11 expression was estimated in cervical cancer tissues and normal tissues in TCGA database. (B) Analysis of IL-11 expression in different clinical stages of TCGA database. (C) IL-11 expression in four cervical cancer cells (HeLa, SiHa, CaSki and C33A) and a normal human cervical epithelial cell line (Ect1/E6E7) was detected by ELISA. Kaplan-Meier survival analyses on different IL-11 expression groups with overall survival (D) and disease free survival (E) in the included 191 cervical cancer patients from TCGA database.

To identify whether IL-11 was upregulated in cervical squamous cell carcinoma, we analyzed data from TCGA, which included 305 cervical cancer tissues and 3 normal tissues. Bioinformatics analysis indicated that IL-11 mRNA was significantly elevated in human cervical cancer tissues compared with normal tissues (Fig. 1A). The expression level of cervical cancer patients was elevated in stage I and Ⅲ compared with normal tissues. Cervical cancer patients in stage II exhibited a higher IL-11 expression than the patients in stage I. However, cervical cancer patients in stage IV exhibited a lower IL-11 expression than the patients in stage I (Fig. 1B). After we detected IL-11 concentrations in the cell supernatants in the cervical cancer cell lines (HeLa, SiHa, CaSki and C33A) and normal cervical epithelial cell lines (Ect1/E6E7) using ELISA analysis. As shown in Fig. 1C, IL-11 concentration was up-regulated in cervical cancer cells (HeLa, SiHa, CaSki and C33A) contrasting with the normal cervical epithelial cell lines (Ect1/E6E7) (Fig. 1C). Kaplan-Meier analysis revealed that patients with high IL-11 expression had a significantly shorter overall survival time with low expression IL-11 (Fig. 1D). In addition, patients with high expression had shorter durations of disease free survival than those with low IL-11 expression (Fig. 1E).

### IL-11 high expression is associated with cervical cancer cell radioresistance

**Figure 2:**
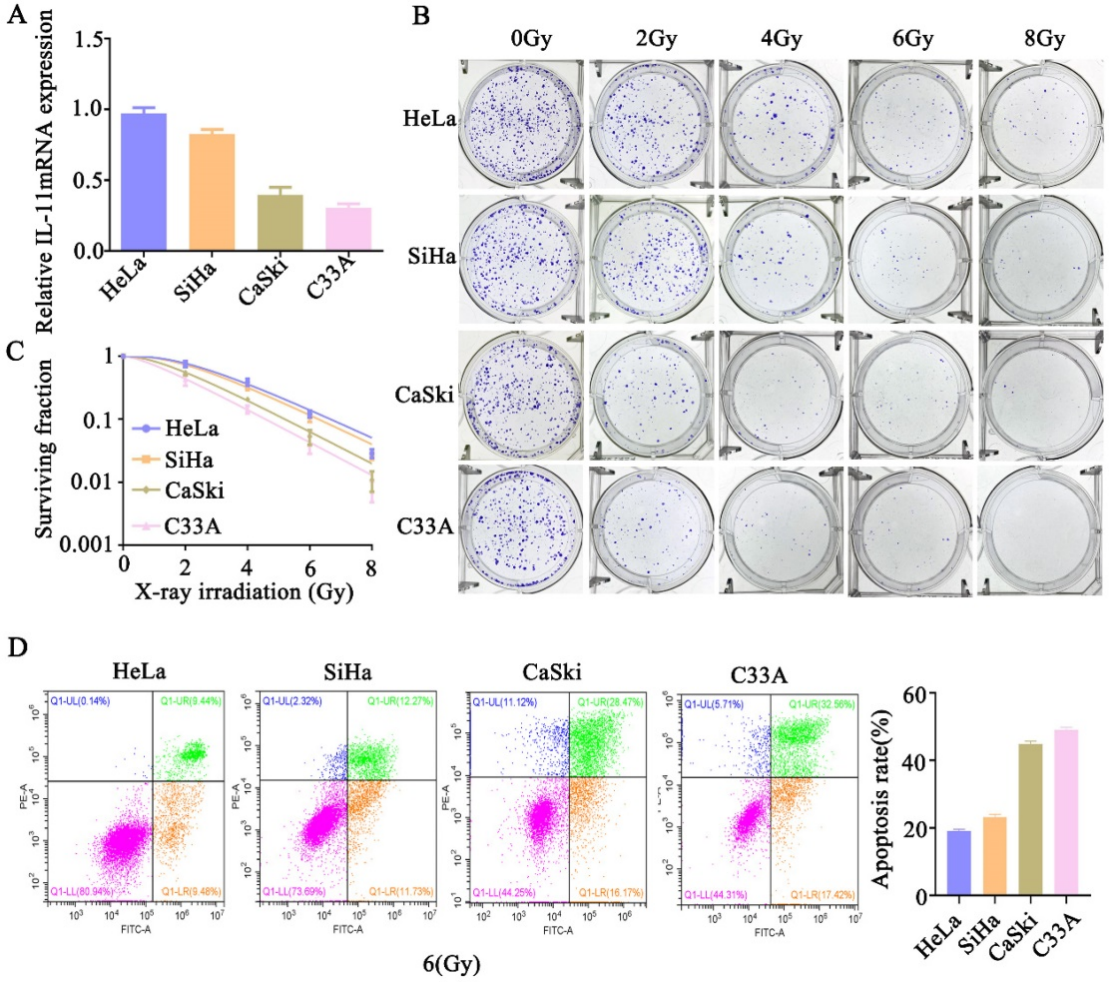
** IL-11 expression is associated with radiation X-ray resistance in cervical cancer.** (A) IL-11expression in HeLa, SiHa, CaSki, C33A were determined by qPCR. (B and C) The effects of X-ray irradiation on cell growth were analyzed by colony formation assay. (D) Cell apoptosis rates were determined by the Annexin V-FITC/PI binding assay 48 h after 6 Gy X-ray irradiation.

We further analyzed the expression level of IL-11 in cervical cancer cells. The qPCR analysis showed the expression of IL-11 mRNA was the highest in HeLa cells, followed by SiHa and CaSki cells, and the lowest in C33A cells (Fig. 2A). We determined the cell survival rate of four cervical cancer cells lines HeLa, SiHa, CaSki and C33A by a standard clonogenic assay, after exposure to various doses of X-ray irradiation (0, 2, 4, 6 and 8 Gy). The results showed that at the same radiation dose, the decreasing trend of cell viability was as follows: HeLa, SiHa, CaSki and C33A (Fig. 2B and C). Additionally, we assess irradiation treatment-induced cell apoptosis by Annexin V-FITC/PI staining using flow cytometry. As shown in Figure 2D, after 6 Gy X-ray irradiation, the increasing trend of apoptotic percentages in cells was as follows: HeLa, SiHa, CaSki and C33A. These findings confirmed that the radioresistance of HeLa cells was lowest among them, meanwhile, c33a cells was highest. These results indicated that IL-11 may be associated with the radioresistance of cervical cancer cells.

### Knockdown of IL-11 enhances cervical cell radiosensitivity

**Figure 3:**
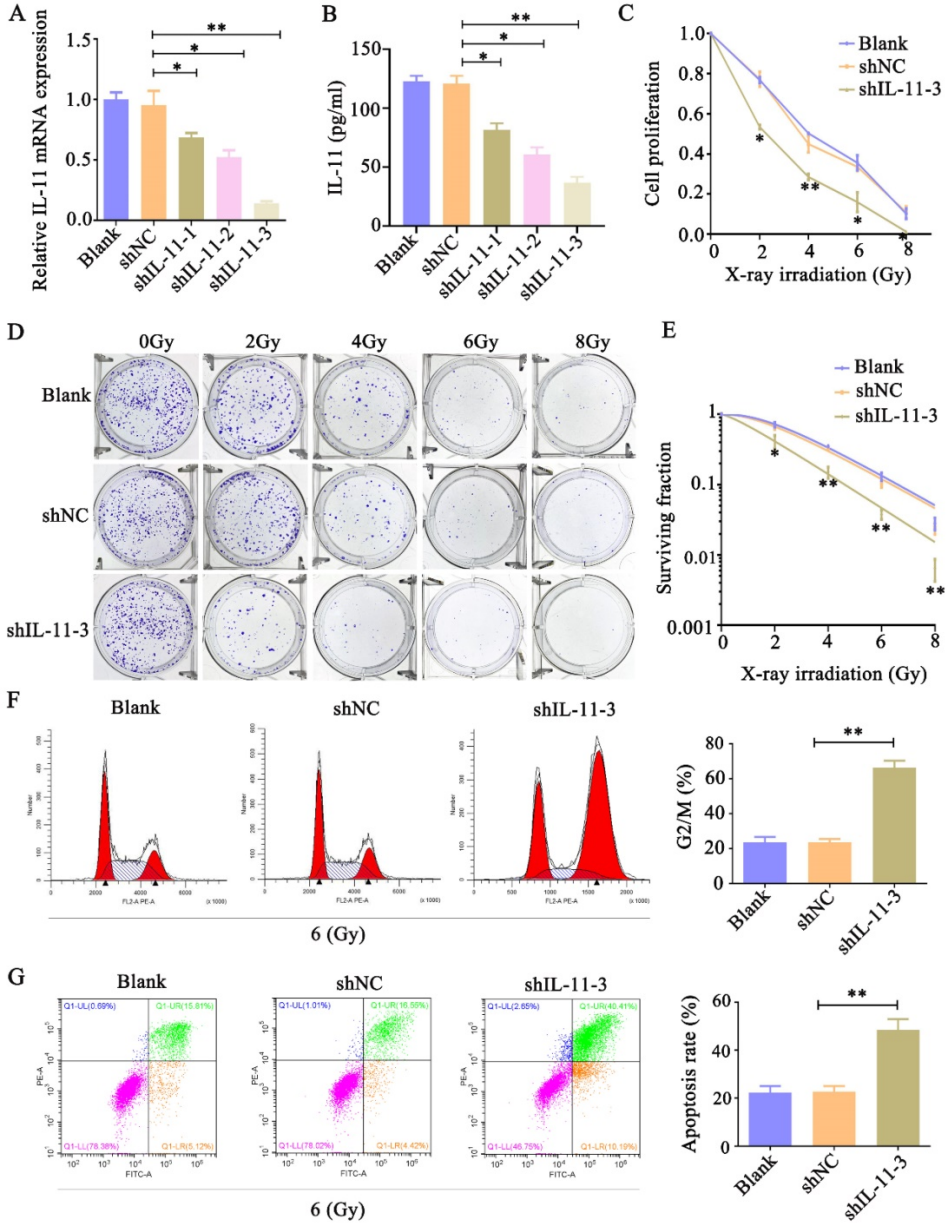
** Knockdown of IL-11 expression enhances the radiosensitivity of HeLa cells *in vitro*.** IL-11 expression in HeLa cells transfected with IL-11shRNA or negative control shRNA was determined by qPCR (A) and ELISA (B). (C) Cell proliferation was detected by CCK-8 assays. (D and E) The effects of X-ray irradiation on cell growth were analyzed by colony formation assay. (F) The cell-cycle phase distribution was analyzed 48h after 6 Gy X-ray irradiation. (G) Cell apoptosis rates were determined by the Annexin V-FITC/PI binding assay 48 h after 6 Gy X-ray irradiation. *P<0.05, **P<0.01.

To analyze whether IL-11 can affect the radiosensitivity of cervical cancer cell lines, we inhibited IL-11 by independent shRNAs. As shown in Figure 3A and B, the qPCR and ELISA analyses indicated that the knockdown effect of shIL-11-3 was the most effective among the designed three shRNAs. Therefore, shIL-11-3 was selected in the present study. The CCK-8 assays revealed that interfered IL-11 expression reduced the proliferative ability of HeLa cells when exposed to various doses of X-ray irradiation (0, 2, 4, 6 and 8 Gy) (Fig. 3C and D). Consistent with this, colony formation assays indicated that knockdown of IL-11 by shRNA-3 could apparently reduce the survival fractions (Fig. 3E). In addition, the cell cycle distribution and cell apoptosis were investigated through flow cytometry. As shown in Figure 3F, the proportion of shIL-11-3 transfected cells arrested at the G2/M phase markedly enhanced following 6 Gy X-ray irradiation. Furthermore, IL-11-knockdown cells caused an increase in the rate of apoptosis when exposed to 6 Gy X-ray irradiation (Fig. 3G). Taken together, these results demonstrated that IL-11 downregulation enhances the radiosensitivity of HeLa cells.

### Exogeneous IL-11 promotes cerivical cell radioresistance

**Figure 4:**
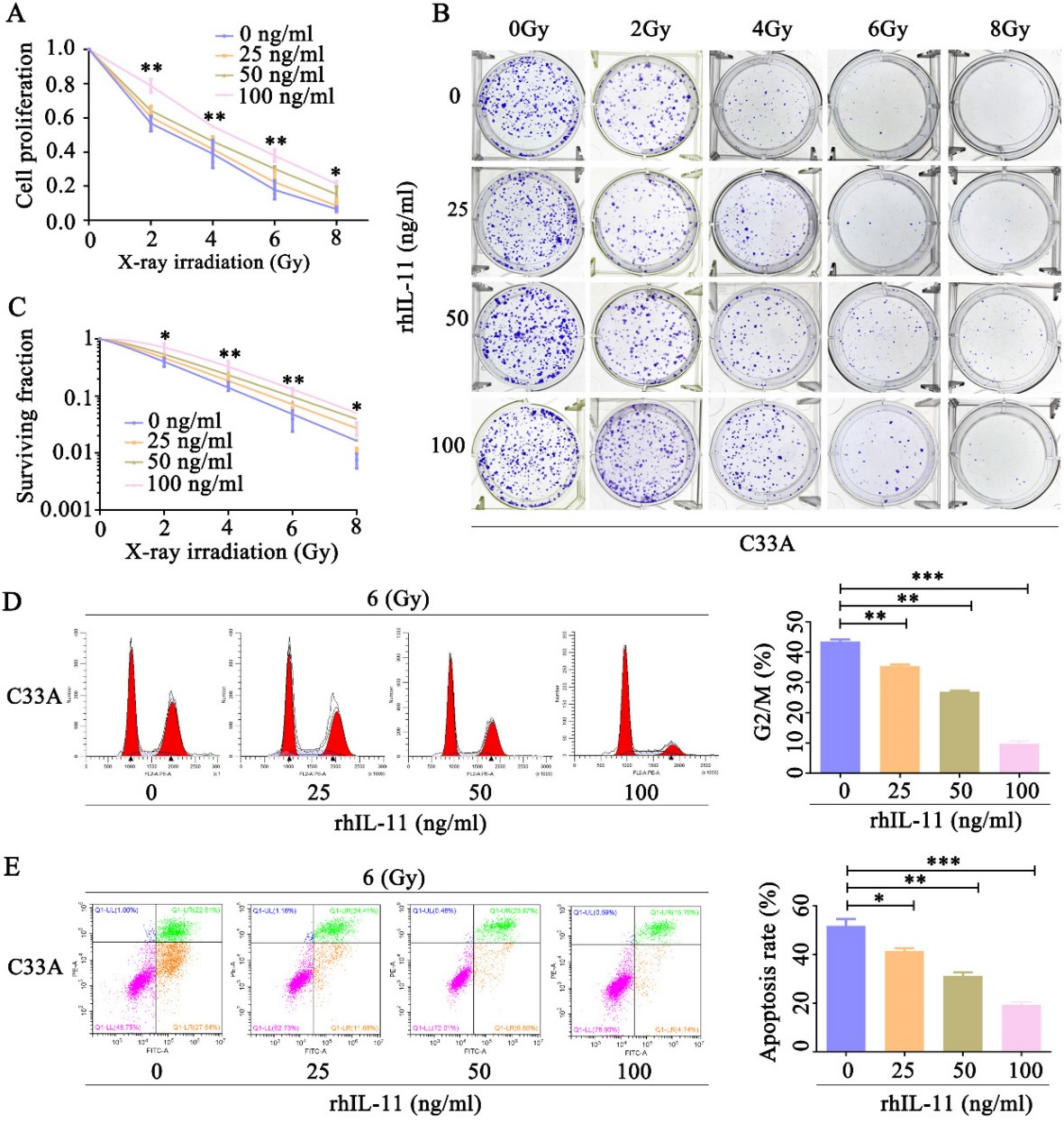
** Exogeneous IL-11 contributes to the radioresistance of C33A cells *in vitro*.** (A) Cell proliferation was detected by CCK-8 assays. (B and C) The effects of X-ray irradiation on cell growth were analyzed by colony formation assay. (D) The cell-cycle phase distribution was analyzed 48 h after 6 Gy X-ray irradiation. (E) Cell apoptosis rates were determined by the Annexin V-FITC/PI binding assay 48 h after 6 Gy X-ray irradiation. *P<0.05, **P<0.01.

Our observation that IL-11-knockdown HeLa cells showed an increased radiosensitivity prompted us to confirm whether exogeneous IL-11 could enhance radioresistance. C33A cells were treated by various concentrations of rhIL-11 (0, 25, 50, 100 ng/ml). By use of CCK-8 assays, we found that rhIL-11 obviously upregulated cell proliferative ability of C33A cells after exposure to X-ray irradiation at doses ranging from 0 to 8 Gy (Fig. 4A). Results of the colony formation assays also indicated that rhIL-11 (25, 50, 100 ng/ml) induced a dramatic increase of clonogenic survival fraction in C33A cells (Fig. 4B and C). The percentage of C33A cells treated by rhIL-11(25, 50, 100 ng/ml) arrested at the G2/M phase markedly decreased after 6 Gy X-ray irradiation (Fig. 4D). Annexin V/PI staining assays showed that rhIL-11 (25, 50, 100 ng/ml) significantly decreased apoptosis rates of C33A cells after exposure to 6 Gy X-ray irradiation (Fig. 4E). We also checked whether regulation of IL-11 expression affects the radiosensitivity of human normal cervical epithelial cells. Ect1/E6E7 cells were treated by various concentrations of rhIL-11(0, 25, 50, 100 ng/ml). The CCK-8 assays revealed that rhIL-11 enhanced cell proliferative ability of Ect1/E6E7 cells after exposure to 6 Gy X-ray irradiation (Fig. S1A). The results of flow cytometry exhibited that the 100 ng/ml of rhIL-11 decreased proportion in the G2/M phase (Fig. S1B) and apoptosis rates of Ect1/E6E7 cells after exposure to 6 Gy X-ray irradiation (Fig. S1C). These results suggest that rhIL-11 could improve the radioresistance of cervical cancer cell C33A and normal cervical epithelial cell Ect1; however, C33A cell is more sensitive to IL-11 than Ect1/E6E7 cell.

### IL-11 activates the PI3K/Akt signaling pathway

**Figure 5:**
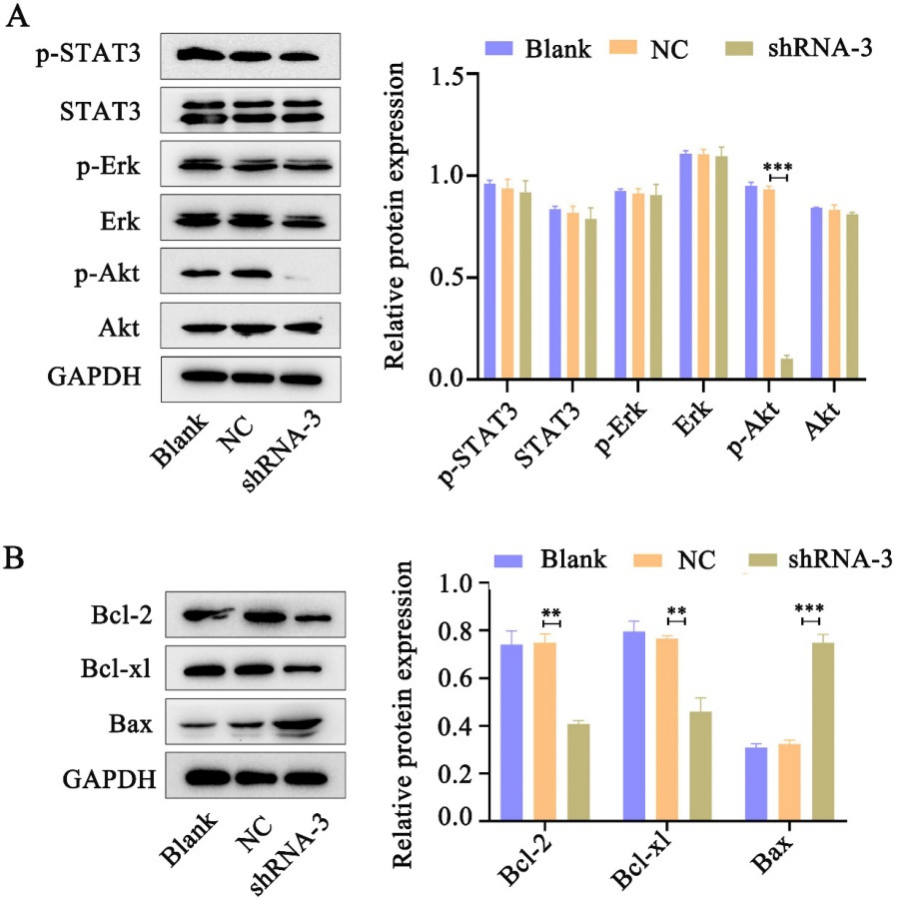
** IL-11 silencing attenuates the PI3K/Akt signaling pathway.** (A) The protein expression of p-STAT3, STAT3, p-Akt, Akt, p-Erk and Erk was detected by western blotting. (B) The protein expression of Bcl-2, Bcl-xl and Bax was detected by western blotting.

Accumulating evidence indicates that IL-11 plays essential roles in oncogenesis through three main signaling pathways: JAK-STAT3, RAS-Erk and PI3K-Akt pathways 12. Western blot analysis was performed to determine pSTAT3, pErk and pAkt levels in HeLa cells in response to IL-11 shRNA treatment. We found that phosphorylation of Akt expression significantly reduced in the IL-11 knockdown Hela cells. However, knockdown of IL-11 expression had no apparent impact on STAT3 and Erk phosphorylation in HeLa cells (Fig. 5A). In addition, we investigated the changes of Bcl-2 (anti-apoptotic protein), Bcl-xl (anti-apoptotic protein), and Bax (pro-apoptotic protein) which represent downstream target genes of Akt signaling pathway. Western blotting revealed that Bcl-2 and Bcl-xl protein levels were downregulated, and the Bax protein levels were upregulated in the IL-11 knockdown Hela cells (Fig. 5B). Our results suggest that IL-11 reduction enhances the radiosensitization of cervical cancer patients may be through inhibiting the PI3K/Akt signaling pathway.

### Inhibition of the PI3K/Akt pathway reversed IL-11-induced radioresistance

**Figure 6:**
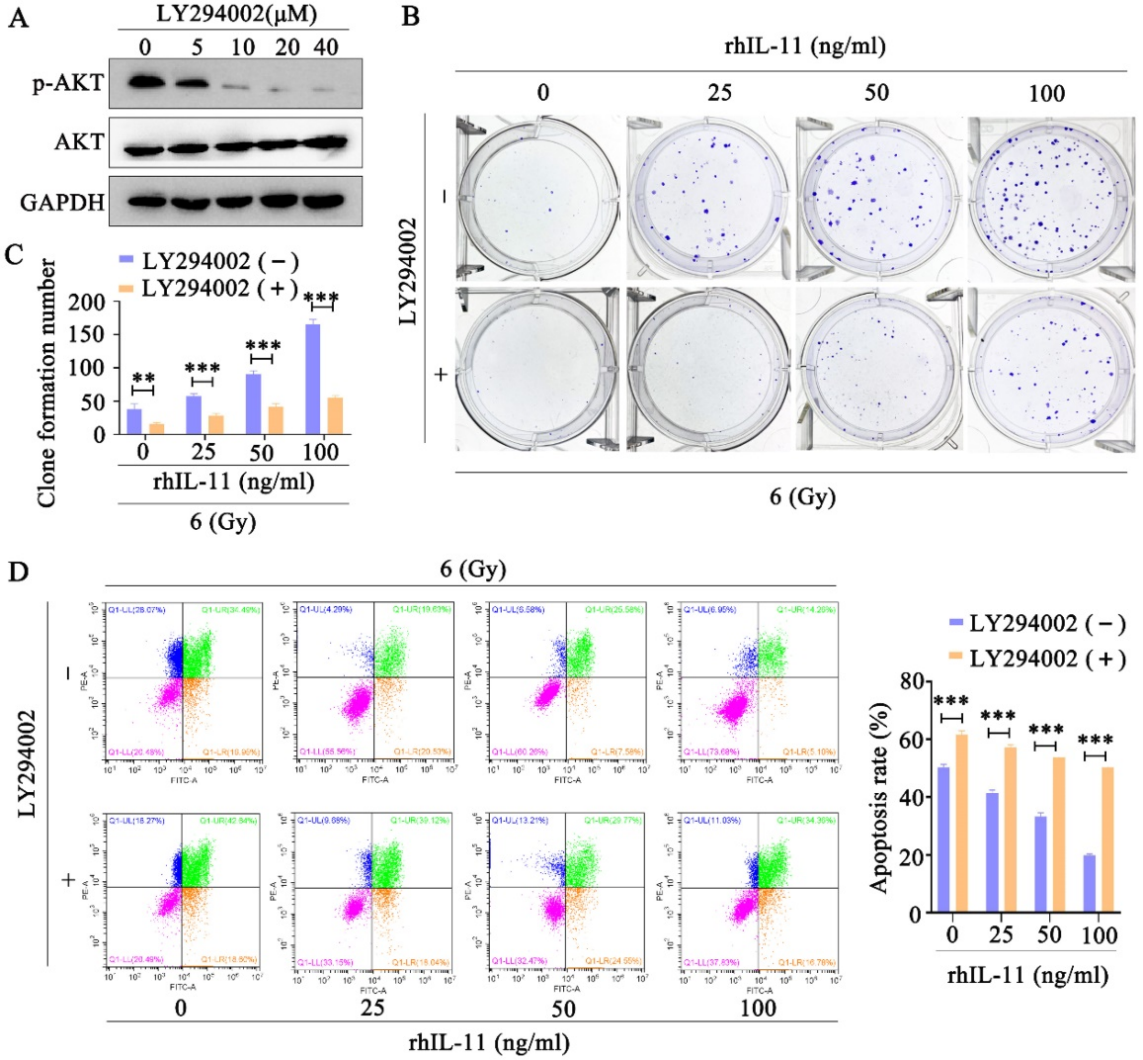
** Inhibition of PI3K/Akt signaling pathway can reverse radioresistance of cervical cancer cells.** (A) The protein expression of p-Akt and Akt was detected by western blotting. (B and C) The effects of 6 Gy X-ray irradiation on cell growth were analyzed by colony formation assay. (D) Cell apoptosis rates were determined by the Annexin V-FITC/PI binding assay 48 h after 6 Gy X-ray irradiation.

To confirm the involvement of PI3K/Akt signaling pathway in IL-11-induced radioresistance, we used the Akt inhibitor LY294002 to inhibit the Akt phosphorylation. The Western blot analyses indicated that Akt phosphorylation was effectively inhibited by LY294002 (10, 20, 40 μΜ) in C33A cells (Fig. 6A). In addition, the Akt inhibitor LY294002 (40 μΜ) significantly decreased the survival fraction (Fig. 6B and C) and promoted apoptosis (Fig. 6C) in C33A treated by rhIL-11 after exposure to 6 Gy X-ray irradiation. Our results indicate that IL-11 causes the radioresistance of cervical cancer through PI3K/Akt signaling pathway.

## Discussion

Radioresistance remains a significant obstacle to limiting the effectiveness of radiotherapy for cervical cancer. Unfortunately, the molecular mechanisms responsible for the radioresistance of cervical cancer are poorly understood. In this study, we found that the expression of IL-11 was increased in radioresistant cervical cancer cells compared to radiosensitive cervical cancer cells. Moreover, we for the first time show that IL-11 influenced radiotherapy tolerance of cervical cancer cells through the PI3K/Akt signaling pathway. Our findings indicate that targeting IL-11 may be a potential strategy to overcome radioresistance in cervical cancer.

IL-11 is a hematopoietic cytokine belonging to the member of the IL-6 family of cytokines, which also includes IL-6, leukemia inhibitory factor (LIF), oncostatin M (OSM), ciliary neurotrophic factor (CNTF), cardiotrophin-1 (CT-1), cardiotrophin-like cytokine (CLC), IL-27 and IL-31. IL-11 could exert pleiotropic effects including stimulating hemopoiesis 13 and thrombopoiesis 14, regulating macrophage differentiation 15 and bone metabolism 16, providing mucosal protection after radiation and chemotherapy 17. Subsequently, there were growing evidences that IL-11 is overexpression in other cancer types, such as gastric cancer, breast cancer, endometrial cancer 18-20, suggesting a critical role of IL-11 in cancer progression. Recent reports have also shown that IL-11 functions as a prominent pro-tumorigenic cytokine through activation of the JAK-STAT3, RAS-ERK and PI3K-Akt pathways 12. Nakayama et al. 18 found that IL-11 promoted the invasive activity of gastric carcinoma cells through the PI3K and MAPK pathways. Zhao et al. 10 claimed that activation Akt and STAT3 signal is associated with the IL-11 induced the tumor growth enhancement in Non-Small Cell Lung Cancer. Ma and his colleagues demonstrated that IL-11 could enhance the chemoresistance of gastric cancer cells by modulating the JAK/STAT3 signaling pathway 21. Notably, a recent study reported that in the absence of Lnk, hematopoietic stem cells (HSCs) become radioresistant, at least partly due to IL-11-mediated activation of both the STAT3 and ERK pathways, indicating that IL-11 may be linked to radioresistance 22. Nevertheless, the exact roles and underlying mechanisms of IL-11 in cervical cancer radioresistance have not been elucidated yet. Our studies demonstrated that IL-11 is overexpressed in radioresistant cervical cancer cells, indicating that IL-11 may be involved in the radiosensitivity of cervical cancer. Subsequent functional studies confirmed this notion that knockdown of IL-11 could increase the radiosensitivity of radioresistant cervical cancer cells, whereas rhIL-11 enhanced the radioresistance in radioresistant cervical cancer cells.

PI3K/Akt signaling has been shown to play essential roles in the regulation of numerous biological processes, including cell proliferation, apoptosis, differentiation, migration, and metabolism 23. Previous studies have found that PI3K/Akt signaling pathway is activated in a wide range of tumors, the activated PI3K/Akt is associated with progression, invasion, metastasis in multiple tumor types 24. Akt activation is associated with radioresistance in some malignant tumors such as head and neck cancer, prostate cancers, non-small cell lung cancer and cervical cancer 25-28. Mechanistically, PI3K-Akt induces radioresistance by enhancing aerobic glycolysis, accelerating repair of IR-induced DNA double-strand breaks (DNADSB), activating tumor-cell proliferation and attenuating radiation-induced apoptosis 27, 29, 30. In addition, an increasing number of studies indicate that inhibition of PI3K/Akt signaling by pharmacological inhibitors could lead to increased radiosensitivity of cancer cells. In this study, we found that IL-11 knockdown cells decreased the expression of p-Akt and the downstream apoptosis-related protein: Bcl-2 (anti-apoptotic protein), Bcl-xl (anti-apoptotic protein), and Bax (pro-apoptotic protein). Pharmacological inhibition the expression of Akt by LY294002 restored the sensitivity of rhIL-11 cells to radiation, as evidenced by less clonogenic survival and enhanced apoptotic response. Accordingly, our work revealed that IL-11 participates in radioresistance in cervical cancer through activating the PI3K/Akt signaling pathway.

In summary, for the first time, we demonstrate that IL-11 is critical for the development of radioresistance in cervical cancer cells. The mechanisms of radiosensitization might be inhibition of the PI3K/Akt signaling pathway. Given that IL-11 is overexpressed and promotes radioresistance in cervical cancer, IL-11 might serve as an attractive candidate to predict radiosensitivity and is a potential therapeutic target for cervical cancer.

## Supplementary Material

Supplementary figure.
