# Supplementary material for: IL-11 mediates the Radioresistance of Cervical Cancer Cells via the PI3K/Akt Signaling Pathway
Source: J Cancer. 2021 Jun 1;12(15):4638–47. doi: 10.7150/jca.56185 (PMC8210555; doi:10.7150/jca.56185)
Supplement: Supplementary file 1 — Supplementary figure. [file jcav12p4638s1.pdf]

Figure S1. Exogeneous IL-11 contributes to the radioresistance of Ect1 cells in vitro. (A) Cell proliferation was detected by CCK-8 assays. (B) The cell-cycle phase distribution was analyzed 48 h after 6 Gy X-ray irradiation. (C) Cell apoptosis rates were determined by the Annexin V-FITC/PI binding assay 48 h after 6 Gy X-ray irradiation. *P<0.05.

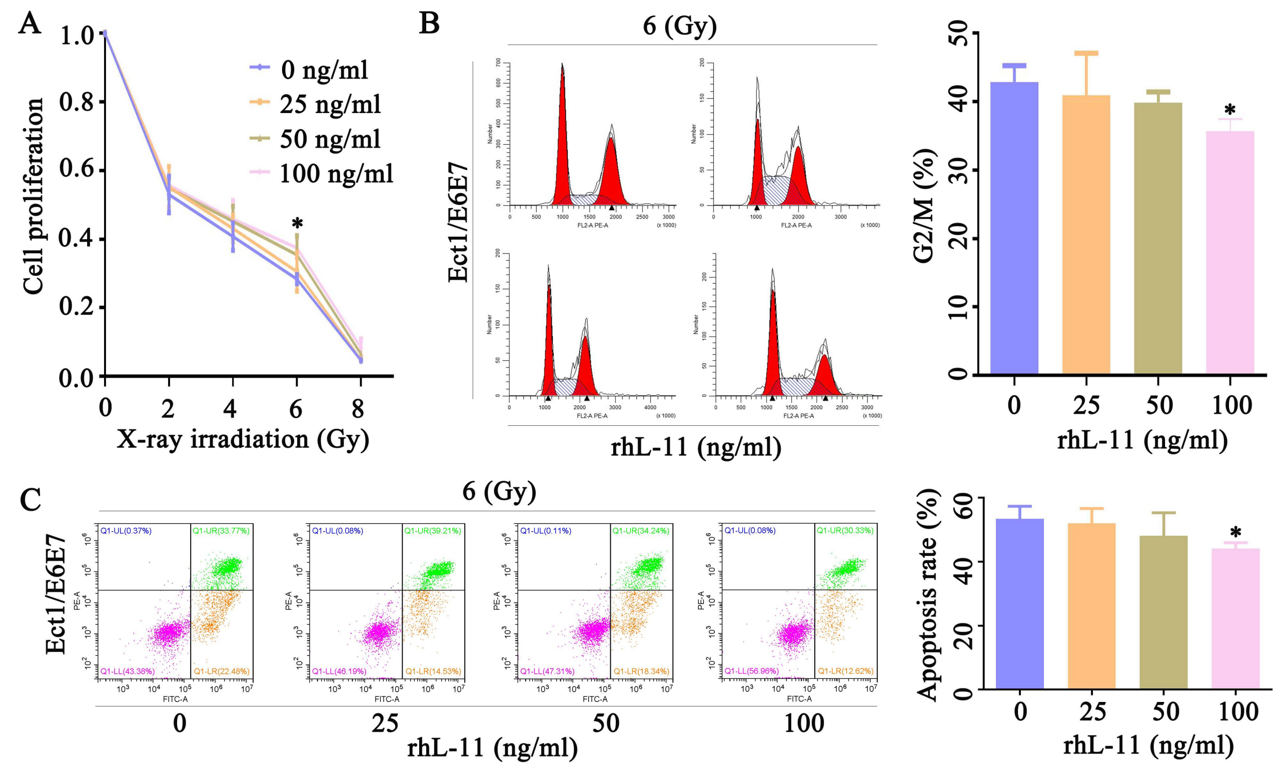
A
B
C
Cell proliferation
X-ray irradiation (Gy)
0 ng/ml
25 ng/ml
50 ng/ml
100 ng/ml
6 (Gy)
Ect1/E6E7
rhL-11 (ng/ml)
G2/M (%)
Apoptosis rate (%)
Q1-UL(0.37%)
Q1-UR(33.77%)
Q1-LL(43.38%)
Q1-LR(22.48%)
Q1-UL(0.08%)
Q1-UR(39.21%)
Q1-LL(46.19%)
Q1-LR(14.53%)
Q1-UL(0.11%)
Q1-UR(34.24%)
Q1-LL(47.31%)
Q1-LR(18.34%)
Q1-UL(0.08%)
Q1-UR(30.33%)
Q1-LL(56.96%)
Q1-LR(12.62%)
FITC-A
PE-A
FL2-A PE-A
Number
